# Rebalancing Immune Interactions within the Brain-Spleen Axis Mitigates Neuroinflammation in an Aging Mouse Model of Alzheimer’s Disease

**DOI:** 10.1007/s11481-025-10177-7

**Published:** 2025-02-07

**Authors:** Anna Flavia Cantone, Chiara Burgaletto, Giulia Di Benedetto, Gabriella Gaudio, Cesarina Giallongo, Rosario Caltabiano, Giuseppe Broggi, Carlo Maria Bellanca, Giuseppina Cantarella, Renato Bernardini

**Affiliations:** 1 Present Address: Department of Biomedical and Biotechnological Sciences, Section of Pharmacology, University of Catania, Catania, Italy; 2 Present Address: Clinical Toxicology Unit, University Hospital of Catania, Catania, Italy; 3 Department of Medical and Surgical Sciences and Advanced Technologies “G.F. Ingrassia”, Division of Hematology, University of Catania, Catania, Italy; 4 Department of Medical and Surgical Sciences and Advanced Technologies “G.F. Ingrassia”, Anatomic Pathology, University of Catania, Catania, Italy

**Keywords:** Neuroinflammation, Immune response, Cytokines, CNS-resident innate immunocytes, Splenocytes, Immune exhaustion

## Abstract

Alzheimer’s disease (AD) is the most common cause of dementia worldwide, characterized by accumulation of amyloid-β protein and hyperphosphorylated tau protein in the brain. Neuroinflammation, resulting from chronic activation of brain-resident innate immune cells as well as enhanced peripheral leukocyte access across the blood–brain barrier, crucially affects AD progression. In this context, TNFSF10, a cytokine substantially expressed in the AD brain, has been shown to modulate both the innate and the adaptive branches of the immune response in AD-related neuroinflammation. In this study, we explored whether a TNFSF10-neutralizing treatment could represent a tool to re-balance the overall overshooting inflammatory response in a mouse model of AD. Specifically, 3xTg-AD mice were treated sub-chronically with an anti-TNFSF10 monoclonal antibody for three months, and were then sacrificed at 15 months. TNFSF10 neutralization reduced the expression of the inflammatory marker CD86, inversely related to levels of the anti-inflammatory marker CD206 in the brain of 3xTg-AD mice, suggesting a switch of microglia towards a neuroprotective phenotype. Similar results were observed in the splenic macrophage population. Moreover, flow cytometry revealed a significant decrease of CD4^+^CD25^+^FOXP3^+^ T regulatory cells as well as reduced number of CD11b^+^LY6C^high^ proinflammatory monocytes in both the brain and the spleen of 3xTg-AD mice treated with anti-TNFSF10 monoclonal antibody. Finally, the treatment resulted in lower count of splenic CD4^+^ and CD8^+^ T cells expressing PD1. The data suggest that TNFSF10 system-targeted treatment effectively restrain overshooting central and peripheral inflammation by rebalancing the overall immune response, mitigating the progression of AD pathology.

**Graphical Abstract:**

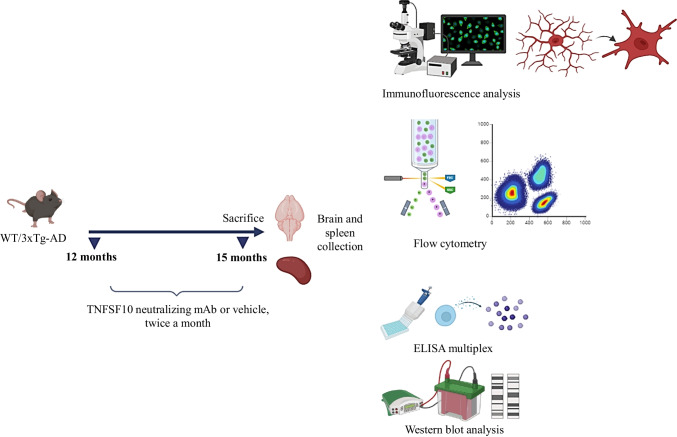

**Supplementary Information:**

The online version contains supplementary material available at 10.1007/s11481-025-10177-7.

## Introduction

Alzheimer's disease (AD) stands as the most prevalent form of age-related dementia, with a rising global incidence, high unmet needs, and limited therapeutic options (Kerwin et al. 2022).

Although a precise cause underlying AD has not yet been defined among an array of potential pathogenetic factors (Vejandla et al. 2024), such as accumulation of amyloid-β (Aβ) and hyperphosphorylated tau (p-tau) in the brain (Gulisano et al. 2018), recently, neuroinflammation has been increasingly regarded as a crucial contributor to AD pathogenesis (Leng and Edison 2021; Uddin et al. 2022; Kiraly et al. 2023).

Neuroinflammation in AD appears associated with chronically activated CNS-resident innate immunocytes (Di Benedetto et al. 2022), as well as with increased re-trafficking of peripheral leukocyte across the blood–brain barrier (BBB) and the choroid plexus (CP) of the blood cerebrospinal fluid barrier (BCSFB) (Zenaro et al. 2017; Gião et al. 2022), supporting the hypothesis of the existence of a bidirectional crosstalk between the inflamed CNS and the peripheral immune system.

Recent findings suggests that the spleen is pivotal in this dialogue (Yu et al. 2021, 2022), as it serves as a major reservoir of immune cells (Lewis et al. 2019).

In this context, robust evidence underscores the critical role of inflammatory/immune mediators in driving the progression of neurodegenerative processes (Chitnis and Weiner 2017; Rani et al. 2023). Among these, TNFSF10, a proapoptotic/proinflammatory cytokine belonging to the TNF superfamily, has been shown to modulate the immune response in AD brain, where it directly mediates Aβ neurotoxicity and, on the other hand, sustains neurodegeneration-related neuroinflammation (Burgaletto et al. 2020).

Cytokine TNFSF10, in fact, is abundantly released by activated glia, CNS-infiltrating macrophages and injured neurons, finally acting as a cell death signal (Cantarella et al. 2003). Consistently, the persistent expression of TNFSF10 appears related with worsening of functional outcomes in animal models of AD (Cantarella et al. 2015). Furthermore, TNFSF10 also fuels inflammation by recruiting peripheral T regulatory (Treg) cells to the brain of the triple transgenic mouse model of AD (3xTg-AD) (Di Benedetto et al. 2019).

We have previously demonstrated that chronic TNFSF10 immunoneutralization by means of a monoclonal antibody results in protective effects on β-amyloid neuronal toxicity in vitro (Cantarella et al. 2003), restores cognitive behavior (Cantarella et al. 2015), reduces Aβ and p-tau proteins accumulation and dampens the inflammatory processes both in the CNS and in the spleen of 3xTg-AD mice (Di Benedetto et al. 2019).

These findings prompted us to further investigate the consequences of the pharmacological neutralization of TNFSF10, in orchestrating the immune response in advanced stage of AD.

To accomplish this objective, we employed the 3xTg-AD, which develop both plaques and tangles in an age-related manner (Oddo et al. 2003), as well as behavioral impairment paralleled with systemic autoimmune/inflammatory disease (Marchese et al. 2014).

We enrolled 3xTg-AD mice at 12 months of age, a timepoint in which these animals exhibit all the hallmarks of the disease, and treated with an anti-TNFSF10 monoclonal antibody, as previously described (Cantarella et al. 2015). Subsequently the immune response and pathological features were assessed.

Our study demonstrates that neutralizing TNFSF10 with an anti-TNFSF10 monoclonal antibody restores balance to the dysregulated immune responses in both the CNS and the periphery. Notably, this effect occurs when the pathology is already well-established, underscoring the potential of targeting TNFSF10 as a therapeutic strategy for addressing immune dysregulation in later stages of the disease.

## Material and Methods

### Mice

Male 3xTg-AD mice [B6129-Psen1tm1MpmTg (APPSwe, tauP30L) 1Lfa/J], which overexpress mutant APP (APPSwe), Psen1 (PS1M146V), and hyperphosphorylated tau (tauP301L), and wild-type mice (B6129SF2/J) were purchased from Jackson Laboratories. The 3xTg-AD mice (Oddo et al. 2003) were generated by co-injecting two distinct transgene constructs encoding human APPSwe and tauP301L (4R/0 N) (controlled by murine Thy1.2 regulatory elements) into single-cell embryos harvested from mutant homozygous PS1M146V knock-in mice. Age matched wild-type mice of mixed genetic background 129/C57BL6 were used as controls. The animals were maintained on a 12-h light/dark cycle in temperature- and humidity-controlled rooms, and food and water were available ad libitum. Animal experiments were conducted in strict accordance with the ARRIVE guidelines and recommendations for the care and use of laboratory animals. All experiments were carried out according to the Directive 2010/63/EU and the Italian law (D.Lgs. 26/2014). The protocols were approved by the Italian Ministry of Health (authorization n. 552/2020-PR).

### Drug Administration and Experimental Groups

The TNFSF10-neutralizing monoclonal antibody (purified rat anti-mouse CD253) and the vehicle (purified rat IgG2ακ isotype control) were obtained from BD Biosciences, Franklin Lakes, New Jersey, USA. All other compounds were of the highest commercial grade available.

Thirty 3xTg-AD and fifteen wild-type mice were enrolled at 12 months of age and three study groups were used: (i) wild-type plus vehicle; (ii) 3xTg-AD plus vehicle (purified rat IgG2ακ isotype control; BD Biosciences); and (iii) 3xTg-AD plus TNFSF10-neutralizing antibody (purified rat anti-mouse CD253; BD Biosciences). Animals were simultaneously randomised to the treatments without taking any other variable into account to avoid bias. Animals (fifteen per experimental group) were administered with a TNFSF10-neutralizing antibody (concentration: 0.05 mg/ml; 200 μl/ mouse; intraperitoneally) or vehicle (concentration: 0.05 mg/ml; 200 μl/mouse; intraperitoneally) (mouse weight = 25 ± 5 g), twice a month (Monday at 12 a.m.) and sacrificed via CO_2_ inhalation or anesthetized and transcardially perfused with ice-cold 0.1 M PBS (pH 7.4) or ice-cold 4% paraformaldehyde (PFA) at 15 months of age, two weeks after the last injection. The treatment with the anti-TNFSF10 monoclonal antibody effectively neutralized TNFSF10 in 3xTg-AD aging mice (see Supplementary Fig. 1).

### Protein Extraction

Brain and spleen samples of 3xTg-AD mice and age-matched wild-type mice were dissected in ice-cold Hank’s balanced salt solution (HBSS: 137 mM NaCl, 5.4 mM KCl, 0.45 mM KH2PO4, 0.34 mM Na2HPO4, 4 mM, NaHCO3, 5 mM glucose; pH 7.4) and then stored at − 80 °C until use. For protein extraction, brain and spleen tissues were homogenized and sonicated in a lysis buffer containing 150 mM NaCl, 50 mM Tris–HCl (pH 7.5), 5 mM EDTA, 1 mM Na3VO4, 30 mM sodium pyrophosphate, 50 mM NaF, 1 mM acid phenyl-methyl-sulphonyl-fluoride, 5 μg/ml aprotinin, 2 μg/ml leupeptin, 1 μg/ml pepstatin, 10% glycerol, and 0.2% TritonTM X-100. After sonication, the homogenates were centrifuged at 14,000 rpm for 10 min at 4 °C and the supernatant was collected. Protein content of the supernatant was quantified according to the Bradford Assay method (Bradford 1976).

### Western Blot Analysis

Equal amounts of protein (30 μg) of hippocampus and spleen extracts were resolved onto SDS-PAGE gels and transferred onto Hybond-ECL nitrocellulose membranes (10600003, Amersham Life Science, Buckinghamshire, UK). Membranes were blocked at room temperature for 1 h with a blocking solution composed of 5% non-fat dry milk (Bio-Rad Laboratories, Segrate, Italy) in phosphate-buffered saline plus 0.05% Tween-20 (PBS-T) and were then probed at 4 °C overnight with the following appropriate primary antibodies: rabbit anti-TNFSF10 (1:200, ab231265, Abcam, Cambridge, UK) or a mouse anti-CD86 (1:500, sc-28347, Santa Cruz Biotechnology Inc., Santa Cruz, CA, USA) or a mouse anti-CD206 (1:500, sc-58986, Santa Cruz Biotechnology Inc.). After that, the membranes were washed with PBS-T and incubated with the appropriate horseradish peroxidase-conjugated secondary antibodies (GE Healthcare) for 1 h at room temperature in 5% non-fat dry milk. For immunodetection, the membranes were exposed to film after enhanced chemiluminescence (ECL) (GE Healthcare). β-actin (1:1000, 4967, Cell Signaling Technology, Danvers, MA, USA.) was used as an internal control to validate the right amount of protein loaded on the gels. Densitometric analysis of band intensity was performed with the aid of ImageJ software version 1.53v (developed by NIH, freeware, available online: https://imagej.nih.gov/ij).

### Flow Cytometry Analysis

Brain tissues were cut with scissors into small pieces on ice and incubated with 0.4 mg ml–1 of collagenase type IV (Gibco, Cat. No. 17104019) for 30 min at 37 °C. After incubation, brain pieces were passed repeatedly through a 19-gauge needle with a syringe to obtain a homogeneous cell suspension, filtered through a 70 μm cell strainer, washed with ice-cold 1 × PBS, and centrifuged at 600 × g for 6 min. The supernatant was aspirated, and 37% Percoll (GE Heathcare, Uppsala, Sweden) was added. The samples were centrifuged at 800 × g for 30 min and the supernatant was discarded, and cell pellet was resuspended into cell staining buffer. Spleen tissues were minced with scissors into a petri dish with HBSS buffer. Excised spleen pieces were transferred and mashed through a 70 μm cell strainer and then washed with PBS to obtain a spleen single-cell suspension (SCS). SCS was centrifuged at 400 × g for 7 min at 4 °C and the supernatant was discarded. The pellet was resuspended in 1 × RBC lysis buffer for 5 min on ice. After washing with ice-cold PBS, the cell suspension was centrifuged at 400 × g for 7 min at 4 °C and the cell pellet was resuspended in PBS. Dead cells were excluded using VioBlue viability dye (Miltenyi Biotec, Bergisch Gladbach, Germany). Frequency of brain and spleen immune cells was analyzed by multicolor FACS analysis using the following fluorochrome-conjugated antibodies: anti-CD25-VioBright FITC (clone REA568, 1:50, 130–120–172, Miltenyi Biotec), or an anti-CD11b-PerCP-Vio700 (clone REA592, 1:50, 130–113–809, Miltenyi Biotec), or an anti-Ly-6C-PE-Vio770 (clone REA796, 1:50, 130–111–918, Miltenyi Biotec), or an anti-CD4-APC-Vio770 (clone REA604, 1:50, 130–119–132, Miltenyi Biotec) or an anti-CD8a-APC-Vio770 (clone REA601, 1:50, 130–120–806, Miltenyi Biotec) or an anti-CD45-VioGreen (clone REA737, 1:50, 130–110–803, Miltenyi Biotec), or an anti-CD279 (PD1)-PE (clone REA802, 1:50, 130–111–953, Miltenyi Biotec), or an anti-P2RY12 (clone S16007D, 1:200, 848003, BD Bioscences). Briefly, 1 × 10^6^ cells were incubated with the antibodies mix for 30 min at RT in the dark and acquired by flow cytometer (Cytomics FC 500, Beckman Coulter). Analysis was performed using CXP Analysis software. Using a sequential gating strategy, the following cell populations were detected within the CD45^+^ immune cells: proinflammatory monocytes were characterized as CD11b^+^LY6C^high^P2RY12^−^ in the brain and as CD11b^+^LY6C^high^ in the spleen, while exhausted T cells were identified as CD4^+^PD1^+^ or CD8^+^PD1^+^.

In brain and spleen samples were also evaluated the frequency of Treg cells (CD4^+^CD25^+^FoxP3^+^). For Tregs evaluation, 1 × 10^6^ cells were fixed and permeabilized with FoxP3/transcription factor staining buffer set (cat. No. 00–5523-00, Thermo Fisher Scientific), and intracellular staining with anti-FoxP3-APC (clone REA788, 1:50, 130–111–679, Miltenyi Biotec) was performed according to the manufacturer's instructions. The results were expressed as percentage. To unequivocally identify the several subpopulations of interest, all gates were controlled using a stain that lacks just one of the fluorescent markers (FMO) allowing accurate gating.

### Immunofluorescence

Mice were deeply anesthetized and intracardially perfused with ice-cold 4% PFA. Brain and spleen samples were collected and fixed overnight in 10% neutral-buffered formalin (Bio-Optica). After overnight washing, samples were dehydrated in graded ethanol and paraffin-embedded, taking care to preserve their anatomical orientation. Five-micrometer-thick sections were serially cutted, mounted on silanized glass slides and air dried. To remove paraffin, slides were immersed in xylene twice, for 10 min, then rehydrated with graded ethanol, 100%, 95%, 70%, and 50%, twice per 10 min each, and transferred to distilled water. Antigens were retrieved in sodium citrate buffer (10 mM sodium citrate, 0.05% Tween-20, pH 6.0) by microwave for 10 min, followed by rinsing with distilled water. For detection of Aβ plaques (6E10), slides were incubated with 70% formic acid for 20 min. The slides were then washed in PBS containing 0.025% Tween-20 (PBST) twice for 5 min each, blocked in 5% BSA for 1 h at room temperature, in a humid chamber. The following primary antibodies were incubated overnight at 4 °C with BSA 1%: goat anti-Iba1 (1:100, NB100-1028, Novus Biologicals), or a mouse anti-CD86 (1:250, sc-28347, Santa Cruz Biotechnology Inc.), or a mouse anti-CD206 (1:500, sc-58986, Santa Cruz Biotechnology Inc.), or a rabbit anti-CD3 (1:100, ab16669, Abcam) or a mouse anti-FoxP3 (1:100, sc-166212, Santa Cruz Biotechnology Inc.) or a mouse anti-β-amyloid (clone 6E10, 1:200, SIG-39320, Covance, Princeton, NJ, USA) or a mouse anti-p-tau (1:100, sc-32275, Santa Cruz Biotechnology Inc). For immunoreactivity and fluorescence detection, after washing in PBS-T three times for 5 min each, sections were incubated with the appropriate fluorescent-labeled secondary antibodies for 1 h at room temperature in the dark: Alexa Fluor 546 donkey anti-goat (A11056, Thermo Fisher Scientific, Inc.), or Alexa Fluor 488 donkey anti-mouse (A21202, Thermo Fisher Scientific), or Alexa Fluor 488 donkey anti-rabbit (A21206, Thermo Fisher Scientific) or Alexa Fluor 546 donkey anti-mouse (A10036, Thermo Fisher Scientific). Finally, for staining of nuclei and stabilization of fluorescent signals, slides were covered in mounting medium with DAPI (F6057, Fluoroshield; Sigma-Aldrich, Milan, Italy) and secured with a coverslip. Images were acquired using a Zeiss Observer.Z1 microscope equipped with the Apotome.2 acquisition system (Zeiss LSM 700, Jena, Germany) with 5x, 20x, and 40 x objectives and were processed using ImageJ software. To quantify immunohistochemical staining, five sections/sample were analyzed. Mean Fluorescence Intensity (MFI) was used to evaluate the intensity of the immunofluorescence signal. Briefly, after converting the images to 8-bit, inverting to grayscale, and using the appropriate threshold function, the positive stained area in the images was measured (Shihan et al. 2021). Colocalization was quantified with ImageJ software using the Colocalization Colormap plugin (Jaskolski et al. 2005). Fluorescent images were converted to 8-bit, and after setting the auto threshold function, the Colocalization Colormap plugin was used to analyze the percentage of immunoreactivity in each image found at the same pixel. Results are presented as correlation index (Icorr).

Plaque size was measured using the Ellipse tool to quantify the area of individual plaques (μm^2^).

To assess the extent of positive immunolabelling, indicated as positive area, images were converted to 8-bit grayscale, and the appropriate threshold function was applied. The percentage of the immunolabelled area was then calculated (Christensen and Pike 2020).

### Cytokine Analysis by Multiplex ELISA

Hippocampal lysates were assayed using the ProcartaPlex™ Mouse Th1/Th2/Th9/Th17/Th22/Treg Cytokine Panel, 17plex (EPX170-26087–901, Thermo Fisher Scientific, Vienna, Austria), according to the manufacturer’s instructions. The concentrations of analytes were detected with the Luminex MAGPIX instrument (Luminex Corporation, Austin, TX). Data were analyzed with xPONENT® software (Luminex Corporation, Austin, TX). Any analyte with a concentration outside the linear range was excluded from the analysis.

### Statistical analysis

Statistical analysis was performed with Prism GraphPad version 9.0. After testing normality distribution of the data, an appropriate one-way analysis of variance (ANOVA) test was applied, and post-hoc Tukey’s multiple comparisons test was used to determine statistical significance. Aβ plaques data were analyzed using a two-tailed Student's t-test. Data were represented as means ± S.E.M. Significance was set at a p value < 0.05 or p < 0.01; shown as *, and ** respectively.

## Results

### Neutralizing TNFSF10 Switches the Functional Phenotype of Microglia in 3xTg-AD Mice

**Fig. 1:**
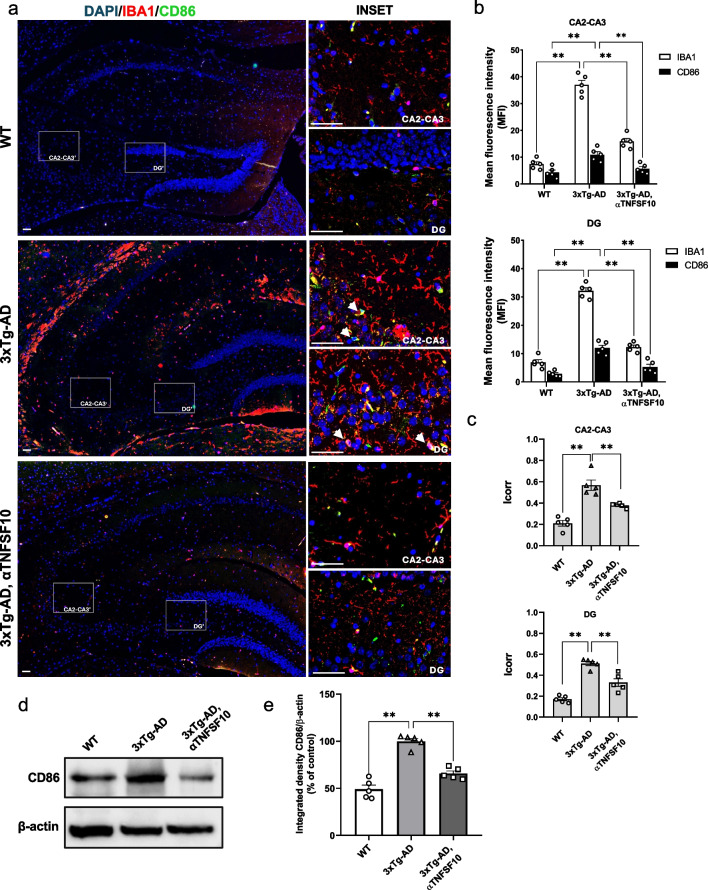
**a** Immunohistochemical detection of Iba-1 and CD86 in the hippocampi of wild-type and 3xTg-AD mice, treated with either vehicle or anti-TNFSF10 mAb. Original magnification 5x; inset 40x. The inserts in photos represent the respective areas magnified (DG = dentate gyrus; CA2-CA3 = cornu ammonis 2–3). Scale bars = 50 µm. **b** Respective mean fluorescence intensity (MFI) analysis of the immunofluorescence signal. **c** Co-localization quantification (Icorr). **d** Western blot analysis of CD86 protein expression in hippocampal lysates of wild-type and 3xTg-AD mice. **e** Respective densitometric analysis of the representative Western blot. Data are expressed as means ± S.E.M. One-way ANOVA and the Tukey’s post hoc test were used to determine statistical significance. **p < 0.01. WT: wild-type (*n* = 5/group); 3xTg-AD mice (*n* = 5/group)

Microglia serve as the primary defense of the CNS in healthy conditions (Colonna and Butovsky 2017). Conversely, in the AD brain, microglia display a pro-inflammatory phenotype, contributing to neuroinflammation and neurodegeneration (Colonna and Butovsky 2017; Gao et al. 2023). To investigate whether neutralization of TNFSF10 could modulate the phenotype of microglia, the expression of Iba1 was evaluated in co-localization with CD86 or CD206 in the brain of 3xTg-AD mice. Immunofluorescence experiments revealed a widespread activation of microglia in the hippocampus of 3xTg-AD mice, as evidenced by the heightened expression of Iba1/CD86 double positive cells, identifying the pro-inflammatory “classically-activated” phenotype. Notably, the expression of both Iba1 and CD86 was markedly reduced in animals treated with the TNFSF10-neutralizing antibody (Fig. 1).

**Fig. 2:**
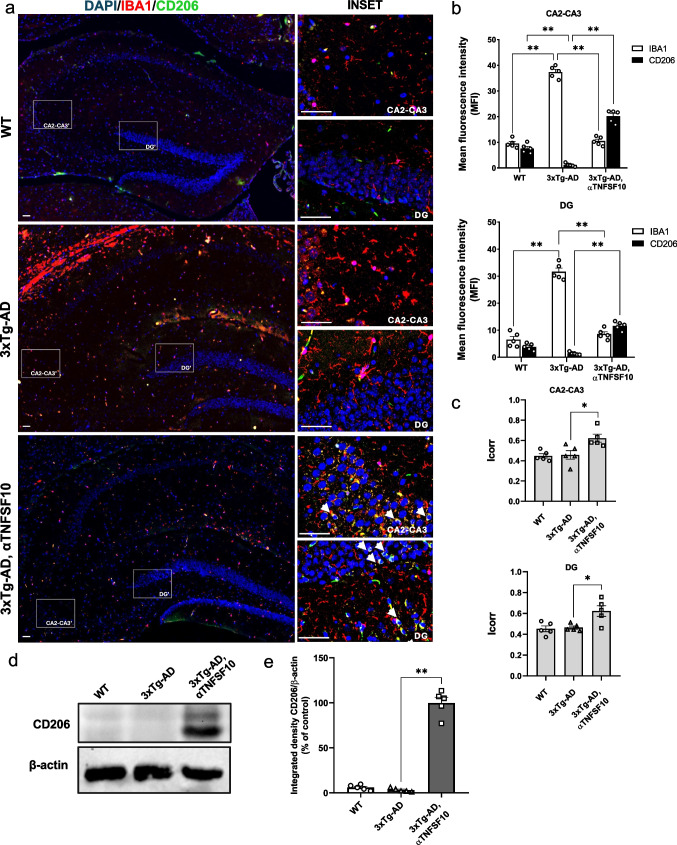
**a** Immunohistochemical detection of Iba-1 and CD206 in the hippocampi of wild-type and 3xTg-AD mice, treated with either vehicle or anti-TNFSF10 mAb. Original magnification 5x; inset 40x. The inserts in photos represent the respective areas magnified (DG = dentate gyrus; CA2-CA3 = cornu ammonis 2–3). Scale bars = 50 µm. **b** Respective mean fluorescence intensity (MFI) analysis of the immunofluorescence signal. **c** Co-localization quantification (Icorr). **d** Western blot analysis of CD206 protein expression in hippocampal lysates of wild-type and 3xTg-AD mice. **e** Respective densitometric analysis of the representative Western blot. Data are expressed as means ± S.E.M. One-way ANOVA and the Tukey’s post hoc test were used to determine statistical significance. *p<0.05, **p < 0.01. WT: wild-type (*n* = 5/group); 3xTg-AD mice (*n* = 5/group)

On the other hand, the expression of Iba1/CD206 double positive cells, which identify the anti-inflammatory “alternatively-activated” microglia, was increased in the hippocampus of 3xTg-AD mice treated with anti-TNFSF10 antibody, compared to untreated 3xTg-AD mice (Fig. 2).

Western blot analysis, consistent with immunohistochemical data, showed a significant decrease in CD86 protein expression and an increase in CD206 protein expression in the hippocampi of anti-TNFSF10 treated 3xTg-AD mice. This suggests a switch of microglia towards a neuroprotective phenotype (Fig. 1 and 2).

### TNFSF10 Immunoneutralization Mitigates 3xTg-AD Brain Inflammation

It is noteworthy that neuroinflammatory foci in the AD brain are linked to glial activation and release of pro-inflammatory cytokines, which have been reported to play a crucial role in AD pathogenesis (Wong-Guerra et al. 2023; Wang et al. 2023).

**Fig. 3:**
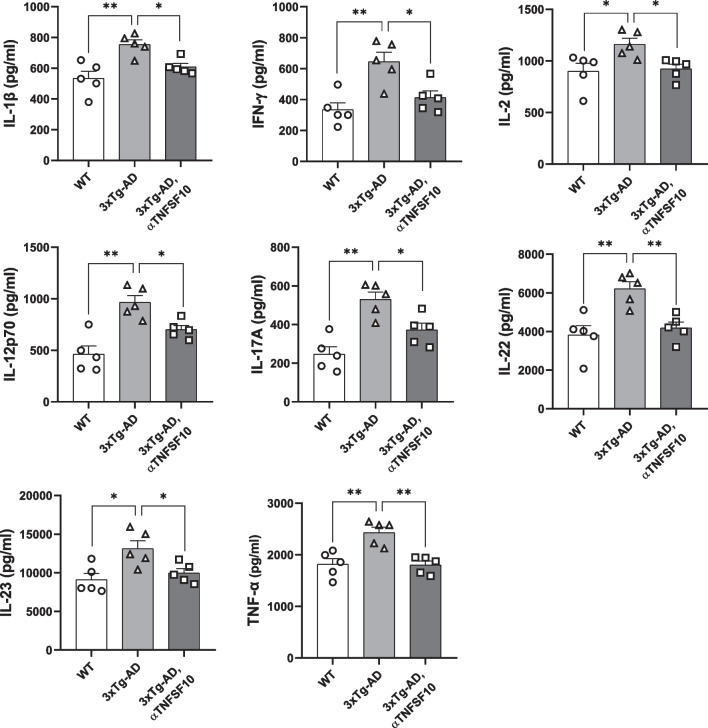
Hippocampal levels of cytokines in wild-type and 3xTg-AD animals receiving either vehicle or an anti-TNFSF10 monoclonal antibody. Cytokines were analyzed by multiplex-ELISA. Values are the mean ± SEM. *p < 0.05, **p < 0.01 by one-way ANOVA followed by Tukey’s post hoc test. WT: wild-type (*n* = 5/group); 3xTg-AD mice (*n* = 5/group)

To better dissect the impact of the anti-TNFSF10 treatment on the neuroinflammatory process in the AD brain, the pattern of pro- and anti-inflammatory cytokines was evaluated by means of multiplex ELISA in 3xTg-AD hippocampal lysates. Specifically, levels of several pro-inflammatory molecules, including IL-1β, IL-2, TNFα, IFN-γ, IL-12p70, IL-17A, IL-22, and IL-23 were significantly elevated in vehicle-treated 3xTg-AD mice. Remarkably, treatment with the TNFSF10-neutralizing antibody restored their levels to those observed in WT mice (Fig. 3).

### Brain Infiltration by Peripheral Inflammatory Monocytes and T Regulatory Cells is Reduced Following Neutralization of TNFSF10

Pro-inflammatory monocytes are among the primary immune cells infiltrating the brain parenchyma following injury, marked by significant BBB disruption, thereby contributing to neuroinflammation (Mildner et al. 2007).

**Fig. 4:**
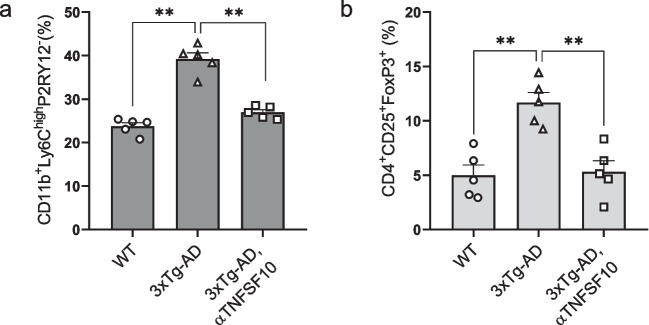
**a** Quantification of the percentage of brain-infiltrating proinflammatory monocytes. **b** Quantification of brain-infiltrating Treg cells. Data are expressed as mean ± SEM. **p < 0.01, (one-way ANOVA followed by Tukey's post hoc test). WT: wild-type (*n* = 5/group); 3xTg-AD mice (*n* = 5/group)

To explore whether TNFSF10 neutralization could result in restraining monocytic-like inflammatory cells, cells isolated from brains, stained with antibodies against either CD45, CD11b, LY6C, and P2RY12 were analyzed by flow cytometry (see Supplementary Fig. 2a for proinflammatory monocytes gating strategy). The frequency (%) of LY6C^high^P2RY12^−^ cells out of the total CD45^+^CD11b^+^ gated cells in the brain of AD mice were significantly augmented compared to WT mice. Remarkably, a pronounced reduction of monocytic-like inflammatory cells was observed in animals underwent the anti-TNFSF10 treatment (Fig. 4 panel a, Supplementary Fig. 2b).

Previous data have shown that TNFSF10 promotes the recruitment of Treg cells (Ikeda et al. 2010) into the brain of 3xTg-AD mice (Di Benedetto et al. 2019). In this context, we investigated whether TNFSF10 neutralization could be associated with reduced infiltration of detrimental Treg cells into the brain.

Isolated brain cells labelled with antibodies against CD45, CD4, CD25, and FOXP3 were analyzed by flow cytometry (see Supplementary Fig. 3a for Treg cells gating strategy). While the percentage of CD25^+^FOXP3^+^ gated cells among the total CD45^+^CD4^+^ in the 3xTg-AD brain were augmented, a significant reduction of the same cells was observed following treatment with anti-TNFSF10 (Fig. 4 panel b, Supplementary Fig. 3b). Indeed, we performed immunofluorescence analysis, corroborating the reduced infiltration of Treg cells in the brains of anti-TNFSF10 treated mice (see Supplementary Fig. 4).

### Neutralization of TNFSF10 Affects Phenotypic Switching of Splenic Macrophages

**Fig. 5:**
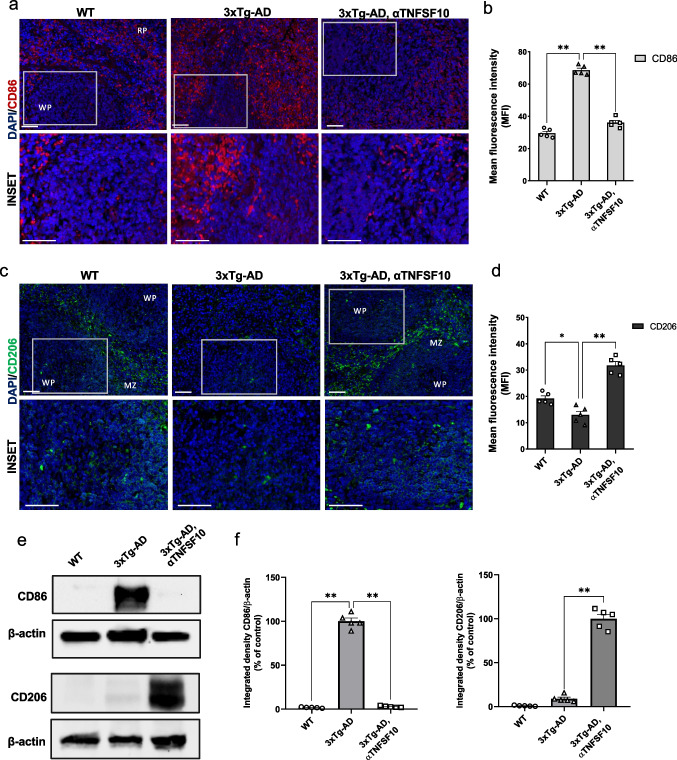
**a** Immunohistochemical detection of CD86 in the spleen of wild-type and 3xTg-AD mice, treated with either vehicle or anti-TNFSF10 mAb. Original magnification 20x; inset 40x. The inserts in photos represent the respective areas magnified. **b** Respective mean fluorescence intensity (MFI) analysis of the immunofluorescence signal. **c** Immunohistochemical detection of CD206 in the spleen of wild-type and 3xTg-AD mice, treated with either vehicle or anti-TNFSF10 mAb. Original magnification 20x; inset 40x. The inserts in photos represent the respective areas magnified. Scale bars = 50 µm. **d** Respective mean fluorescence intensity (MFI) analysis of the immunofluorescence signal (RP = red pulp; WP = white pulp; MZ =marginal zone). **e** Western blot analysis of CD86 and CD206 protein expressions in splenic lysates of wild-type and 3xTg-AD mice. **f** Respective densitometric analysis of the representative Western blot. Data are expressed as means ± S.E.M. One-way ANOVA and the Tukey’s post hoc test were used to determine statistical significance. *p < 0.05; **p < 0.01. WT: wild-type (*n* = 5/group); 3xTg-AD mice (*n* = 5/group)

Recent evidence suggests a potential involvement of the spleen in AD pathogenesis (Wei et al. 2022). Considering that the spleen, serving both as a blood filter and an immune organ, represents a major source of peripheral immune cells (Bronte and Pittet 2013; Lewis et al. 2019), we studied the effect of TNFSF10 neutralization on splenic cell populations. In particular, immunofluorescence and Western blot analysis performed on spleen samples revealed increased pro-inflammatory ‘classically-activated’ macrophages in vehicle-treated 3xTg-AD mice, identified by the macrophage marker CD86. Neutralization of TNFSF10 determined a significant attenuation of CD86-expressing macrophages. Moreover, while the anti-inflammatory macrophage marker CD206 was scarcely measurable in the spleen of untreated AD mice, robust expression was detected in animals that underwent the anti-TNFSF10 treatment (Fig. 5).

### Neutralization of TNFSF10 Reduces the Splenic Populations of both Inflammatory Monocytes and Regulatory T Cells

Several studies have underscored the spleen as the principal reservoir of inflammatory monocytes from which these cells are mobilized during chronic inflammation (Swirski et al. 2009; Robbins et al. 2012; Rizzo et al. 2020).

**Fig. 6:**
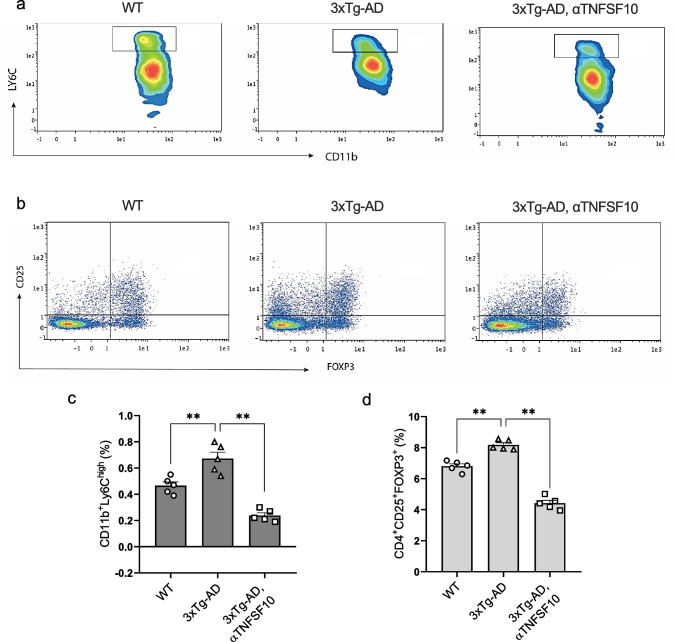
Representative flow cytometry plots for each experimental group used to identify Ly6C^high^ cells out of the total CD11b^+^ gated cells. **b** Representative flow cytometry plots for each experimental group used to identify CD25, FOXP3 positive cells out of the total CD45^+^CD4^+^ gated cells. **c** Quantification of the percentage of splenic proinflammatory monocytes. **d** Quantification of spleen-T reg cells. Data are expressed as mean ± SEM. **p < 0.01 (one-way ANOVA followed by Tukey's post hoc test). WT: wild-type (*n* = 5/group); 3xTg-AD mice (*n* = 5/group)

In order to explore whether neutralization of TNFSF10 could modulate the splenic inflammatory monocytes, splenocytes stained with antibodies against either CD45, CD11b and LY6C were analyzed by means of flow cytometry. Frequency (%) of pro-inflammatory monocytes expressing both CD11b and LY6C^high^ out of the total CD45^+^ gated cells was significantly higher in 3xTg-AD spleens, compared to WT spleens. Indeed, the percentage of splenic CD11b^+^ LY6C^high^ monocytes was significantly lower in 3xTg-AD anti-TNFSF10 treated mice (Fig. 6 panel a).

We have previously shown that Treg cells markers such as GITR and FoxP3, detectable in the spleen of 3xTg-AD mice, are blunted in the spleen of AD mice following the treatment with TNFSF10 neutralizing monoclonal antibody (Di Benedetto et al. 2019). To corroborate the hypothesis that such reduction could correlate with the depletion of Treg in the spleen of 3xTg-AD mice, splenocytes labelled with antibodies against CD45, CD4, CD25, and FOXP3 were analyzed by flow cytometry. Results unveiled that the percentage of CD25^+^FOXP3^+^ T reg cells of the total CD45^+^CD4^+^ T cells in the spleen was significantly depleted in AD mice treated with the anti-TNFSF10 antibody (Fig. 6panel b).

### Neutralization of TNFSF10 Counteracts Peripheral Immune Exhaustion in 3xTg-AD Mice

Among T cells dysfunction, T cell exhaustion, marked by upregulated inhibitory receptors, potentially contributes to pathogenesis and progression of AD (Miggelbrink et al. 2021; Grayson et al. 2023).

Therefore, with the aim to understand whether treatment with anti-TNFSF10 monoclonal antibody could affect immune exhaustion, the expression of either CD4^+^PD1^+^ T cells as well as CD8^+^PD1^+^ T cells was studied in spleen samples of vehicle- and anti-TNFSF10-treated 3xTg-AD mice by means of flow cytometry.

**Fig. 7:**
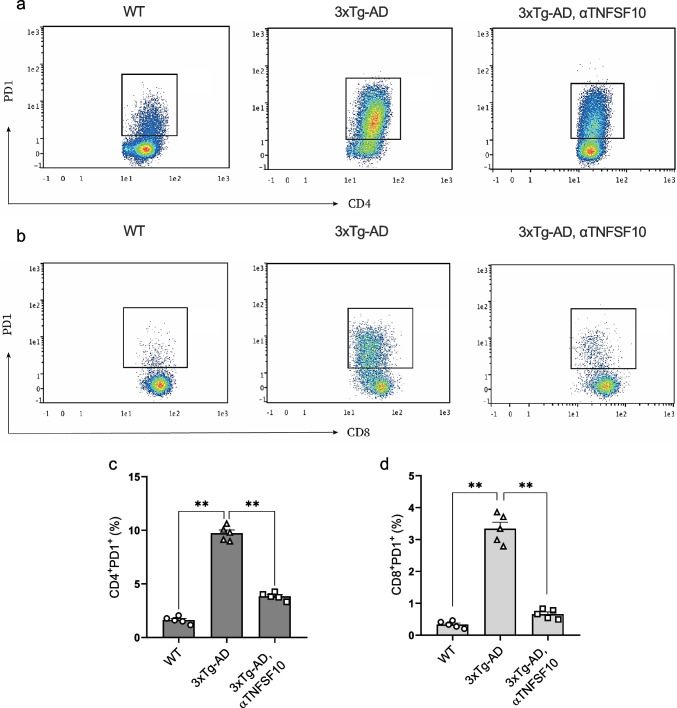
**a** Representative flow cytometry plots for each experimental group used to identify CD4^+^ T cells expressing PD1^+^. **b** Representative flow cytometry plots for each experimental group used to identify CD8^+^ T cells expressing PD1^+^. **c** Quantification of the percentage of splenic CD4^+^PD1^+^ cells out of the total CD45^+^ gated cells. **d** Quantification of the percentage of splenic CD8^+^PD1^+^ cells out of the total CD45^+^ gated cells. Data are expressed as mean ± SEM. **p < 0.01, (one-way ANOVA followed by Tukey's post hoc test). WT: wild-type (*n* = 5/group); 3xTg-AD mice (*n* = 5/group)

Results demonstrated that the frequency of either CD4^+^PD1^+^as well as CD8^+^PD1^+^ cells out of the total CD45^+^ gated cells were markedly augmented in the spleen of vehicle-treated AD mice. Interestingly, in mice treated with anti-TNFSF10, we observed a striking reduction in both PD1-expressing CD4^+^ T cells and PD1-expressing CD8^+^ T cells, confirming that neutralization of TNFSF10 effectively counteracts peripheral immune exhaustion in 3xTg-AD mice (Fig. 7).

### The Anti-TNFSF10 Treatment Reduces Amyloid-Beta Plaques and Phosphorylated Tau Load in 3xTg-AD Mice

In the 3xTg-AD model, similar to human AD brain, Aβ plaques develop in an age-dependent manner, accumulating in AD-relevant brain regions, including the hippocampus, cortex, and amygdala (Oddo et al. 2003; Ono et al. 2024).

Given the concurrent immunological changes and neuropathology observed in 3xTg-AD mice (Marchese et al. 2014), we investigated whether administering anti-TNFSF10 mAb at a late stage of pathology could modulate amyloidosis in both the hippocampus and spleen of these mice.

**Fig. 8:**
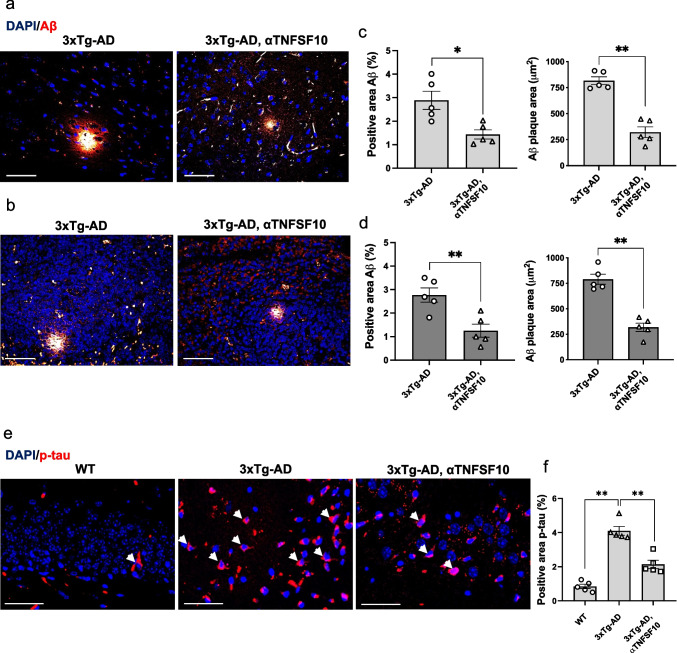
**a** Immunohistochemical detection of Aβ plaques in the brain (**a**) and the spleen (**b**) of 3xTg-AD mice, treated with either vehicle or anti-TNFSF10 mAb. Original magnification 40x. Scale bars = 50 µm. Respective positive staining area and sizes analysis of brain (**c**) and spleen (**d**) Aβ plaques. Data are expressed as means ± S.E.M. *p < 0.05; **p < 0.01 (Two-tailed Student's t-test). **e** Immunohistochemical detection of p-tau in the brain of wild-type and 3xTg-AD mice, treated with either vehicle or anti-TNFSF10 mAb. Original magnification 40x. Scale bars = 50 µm. **f** Respective positive staining area analysis of the immunofluorescence signal (one-way ANOVA followed by Tukey's post hoc test). *p<0.05; **p < 0.01. WT: wild-type (*n* = 5/group); 3xTg-AD mice (*n* = 5/group)

Immunofluorescence staining revealed extensive Aβ plaques accumulation in both organs of vehicle-treated 3xTg-AD mice. In contrast, 3xTg-AD mice subjected to the anti-TNFSF10 mAb treatment exhibited a significant reduction in plaque burden, evidenced by decreased total Aβ immunostaining and smaller plaque size (μm^2^) in both the brain and the spleen (Fig. 8 panel a-d).

Elevated tau phosphorylation widely recognized as pathological hallmarks of AD (Rawat et al. 2022), are closely associated with disease severity and progressively increase in advanced stages (Telser et al. 2023). In this study, we examined whether administering anti-TNFSF10 mAb at advanced stages of pathology could modulate p-tau levels. Immunofluorescence analysis showed that while p-tau expression was markedly heightened in the brain of vehicle-treated 3xTg-AD mice, its expression became greatly attenuated after the treatment with the anti-TNFSF10 mAb, as shown in Fig. 8 panel e, f.

## Discussion

Our previous studies have highlighted a significant role of the proapoptotic/proinflammatory cytokine TNFSF10 in sustaining neuroinflammation in the 3xTg-AD mice. The prominent toxic effects of TNFSF10, which synergizes with other cytokines, are likely due, at least in part, to an imbalanced immune response triggered by Aβ accumulation in both the CNS and in the spleen (Cantarella et al. 2015; Di Benedetto et al. 2019). Consistently, the TNFSF10 neutralizing antibody improved cognitive function (Cantarella et al. 2015), while reducing pathological protein accumulation, and inflammation in 3xTg-AD mice when administered during an early phase of AD (Di Benedetto et al. 2019). However, this time schedule did not fully reflect the clinical reality of AD, where diagnosis is attained once cognitive symptoms are already evident (Tarawneh and Holtzman 2012).

In the wake of these observations, the present study aims to investigate whether administering a TNFSF10-neutralizing mAb at an advanced stage of AD, which more closely mimics the timeline of current clinical practice, could still provide beneficial effects. Specifically, we explored whether such treatment could effectively rebalance the dysregulated central and peripheral immune responses as well as the altered re-trafficking of immune cells between these domains in the 3xTg-AD mouse model. In the AD brain, microglial cells, activated by Aβ plaque accumulation and dying neurons, tend to adopt a detrimental “classically-activated” phenotype (Mandrekar-Colucci and Landreth 2010; Miao et al. 2023), releasing inflammatory mediators including IL-1β, IL-6, TNF-α (Smith et al. 2012) and TNFSF10 (Huang et al. 2005). Blocking pro-inflammatory cytokine signalling has been shown to attenuate microgliosis (Kitazawa et al. 2011; Tweedie et al. 2012; Ou et al. 2021).

Consistently, the anti-TNFSF10 treatment induced dampening of “M1-like” proinflammatory microglia in the hippocampus of 3xTg-AD mice, alongside with an increased expression of “M2-like” anti-inflammatory microglia, suggesting its beneficial impact on microglia phenotype.

As mentioned above, pro-inflammatory cytokines released by CNS-resident immune cells critically fuel AD by damaging neurons and other glial cells and compromising BBB integrity (Merlini et al. 2018; Yang et al. 2022; Zang et al. 2022; Zhang et al. 2023a), while peripherally released cytokines that cross the BBB exacerbate brain inflammation through direct neurotoxicity and activation of microglia and astrocytes (Majerova et al. 2019; Burgaletto et al. 2020). The restraint of the immune response achieved in the CNS through the anti-TNFSF10 treatment was paralleled by a significant decrease in several pro-inflammatory cytokines in the brain of anti-TNFSF10 treated 3xTg-AD mice. These findings support the hypothesis that neutralization of TNFSF10 could dampen the overshooting neuroinflammatory response taking place in the AD brain.

Although the substantial involvement of brain-resident immune cells in the pathogenesis of AD is historically acknowledged, the role of the peripheral immune system remains still largely debated (Giménez-Llort et al. 2012). Under normal conditions, peripheral immune cells are rarely detected in the brain parenchyma (Prinz and Priller 2017). However, growing evidence suggests that peripheral immune cells, including monocytes, NK and T cells, can be actively recruited into the CNS, contributing to AD progression by shaping the inflammatory response (Mrdjen et al. 2018; Castellani and Schwartz 2020; Ennerfelt and Lukens 2020). Thus, in light of such body of evidence which indicates dysregulation in both central and peripheral immune compartments as a key feature of AD, it is becoming increasingly clear how AD might be recognized as the neurological manifestation of a systemic disease (Bettcher et al. 2021).

To corroborate this hypothesis, we here demonstrated a significant infiltration of a Ly6C^high^ population of monocytes, recognized as pro-inflammatory (Li et al. 2022), as well as of Treg cells in the brain of vehicle-treated 3xTg-AD mice, which dramatically decreased following anti-TNFSF10 mAb treatment.

Whilst robust evidence indicates that circulating proinflammatory Ly6C^high^ monocytes are actively recruited by brain-derived inflammatory cues and contribute to inflammatory responses, by releasing inflammatory molecules such as IL-1β and TNF-α (Auffray et al. 2009; Ginhoux and Jung 2014), the role of Treg cells in modulating inflammatory responses after CNS injury is still poorly understood and, at any rate, controversial (Walsh et al. 2014; Duffy et al. 2018; Rocamora-Reverte et al. 2021). The stability of Treg cells in AD has long been debated, as they are suggested to play a beneficial role in early stages of AD by regulating the immune response and, somehow, maintaining an immune equilibrium (Dansokho et al. 2016; Jafarzadeh et al. 2023). However, in later stages of the disease, when embedded in an increasingly inflammatory milieu, Tregs may become unstable and dysfunctional, hindering Aβ clearance, thereby worsening the disease. Furthermore, when their number is significantly increased in the periphery in concomitance to partial disruption of the BBB, Tregs may gain enhanced trafficking to/from the brain parenchyma, contributing to the pathological environment of late-stage AD (Baruch et al. 2015).

Considering TNFSF10 ability to enhance the presence and the activity of Treg cell subsets during an overshooting response (Ikeda et al. 2010), the reduced Tregs infiltration achieved by the anti-TNFSF10 mAb treatment implies that targeting TNFSF10 could be a valuable strategy to modulate Tregs recruitment and accumulation, detrimental in later stages of the pathology.

Additionally, recent, compelling evidence suggests that the brain-spleen axis could be involved in AD progression (Unger et al. 2020; Pakravan et al. 2021; Yu et al. 2022; Wei et al. 2022). Indeed, in vivo studies have shown that the spleen not only reflects the inflammatory status of the AD brain (Di Benedetto et al. 2019), but also serves as reservoir of peripheral immune cells. Specifically, 3xTg-AD mice develop splenomegaly (Marchese et al. 2014) and display abundant Aβ deposits in the spleen, underpinning an impairment of the peripheral immune response in AD (Di Benedetto et al. 2019).

Approximately forty to sixty percent of the brain-derived Aβ flows into peripheral organs for clearance (Roberts et al. 2014; Xiang et al. 2015) with splenic monocytes/macrophages actively uptake Aβ (Yu et al. 2022), based also on their ability to adopt either proinflammatory or “classically-activated” and anti-inflammatory or “alternatively-activated” phenotypes (Mulder et al. 2014).

We found heightened expression of splenic “classically-activated” macrophages in 3xTg-AD mice, significantly attenuated after anti-TNFSF10 mAb treatment. On the other hand, the treatment induced an increase in “alternatively-activated” macrophages.

The spleen has been identified as an alternative reservoir of Ly6C^high^ monocytes, readily recruitable to exacerbate inflammation at distant sites (Swirski et al. 2009; Robbins et al. 2012). In this line, our study further supports the role of the spleen as a source of Ly6C^high^ monocytes in the AD pathology, with a highly represented proinflammatory Ly6C^high^ monocytes population in the spleen of 3xTg-AD mice, reduced upon anti-TNFSF10 mAb treatment.

Although the role of spleen-derived Treg cells in this context is not fully elucidated, our experiments revealed that these cells were highly represented in the spleen of 3xTg-AD mice. It appears plausible to hypothesize that the spleen could serve as a source of excess Tregs, especially considering its enlarged size (Romano et al. 2016; Di Benedetto et al. 2019) and hyperactivity found in 3xTg-AD mice (Romano et al. 2016; Di Benedetto et al. 2019). Consistently, the increased number of brain Tregs likely reflects their TNFSF10-mediated abundance in the spleen, and the TNFSF10-related Treg cell-depleting effect in the latter, would imply their decrease across all organs, including the brain.

Thus, not only our observations are well aligned with the changes of the infiltrated peripheral immune cells occurring in the brain of these animals, but also provide evidence that TNFSF10 neutralization, by restraining inflammatory cues present within their microenvironment, is associated with a rebalanced brain-spleen trafficking of immunocytes.

The suppression of the overall inflammatory response achieved by means of the anti-TNFSF10 mAb treatment is also accompanied by a significant depletion of splenic CD4^+^ and CD8^+^ T cells expressing high level of PD1, an inhibitory immune checkpoint receptor that dampen T cell activation and function (Miggelbrink et al. 2021).

Emerging studies suggest that inhibiting PD1 signaling can improve clearance of cerebral Aβ plaques, cognitive performance and enhance peripheral immune responses (Baruch et al. 2016; Deczkowska and Schwartz 2018), supporting the hypothesis that targeting exhausted T cells could represent a viable treatment option for AD.

Although the exact underlying mechanism is not yet fully understood, TNFSF10 neutralization, while beneficially affects the inflammatory environment, reducing proinflammatory monocytes and detrimental Tregs in the spleen, could, at the same time, modulates T cells function. Indeed, chronic exposure to inflammatory signals (i.e. IFNs and IL-6) drives T cell exhaustion (Yi et al. 2010; Wherry and Kurachi 2015) and improving the inflammatory milieu, may help reverse this effect (Yi et al. 2010; Saeidi et al. 2018).

Given that Aβ plaques deposition is a feature typical of AD pathogenesis and progression (Zhang et al. 2023b), and in light of the changes in the immune environment observed both in the brain and the spleen, we investigated whether the anti-TNFSF10 mAb treatment could affect amyloidosis in 3xTg-AD mice. We previously demonstrated that TNFSF10 neutralization in the early stages of the disease dramatically halted Aβ deposition in both the hippocampus and the spleen of the same strain (Di Benedetto et al. 2019). Here, we demonstrated that anti-TNFSF10 mAb administration in aged 3xTg-AD mice concur to a notable reduction of Aβ plaque deposition in both organs of these mice. This effect might be mediated by the enhancement of brain microglia and splenic macrophages' Aβ-phagocytic function, potentially based on the ability of the treatment to modulate their phenotypes, eventually inducing a rebalance of the immune response.

The improvement in brain pathology observed in anti-TNFSF10 mAb-treated 3xTg-AD mice was accompanied by a significant reduction in p-tau protein levels. Indeed, Aβ aggregates have been shown to promote tau hyperphosphorylation, establishing a direct link between Aβ and tau in causing synaptic dysfunction and neuronal damage, which are pivotal contributors to toxicity in AD (Ittner and Götz 2011; Rawat et al. 2022). These findings underscore the potential of TNFSF10 neutralization as a promising strategy to halt brain pathology, even in advanced stages, by modulating central and peripheral immune response.

In this study, we focused on male mice to minimize variability in inflammatory responses, which are influenced by hormonal fluctuations and other sex-specific factors in female models (Murtaj et al. 2019; Dennison et al. 2021). While this approach allowed us to minimize potential confounding variables, we acknowledge the critical importance of addressing sex differences in neurodegenerative diseases, including AD, where women exhibit higher prevalence rates and distinct inflammatory profiles (Lopez-Lee et al. 2024). Therefore, while our findings provide valuable insights, extrapolating these results to women requires careful consideration of these sex-specific immune responses. Future studies including female models will be crucial to further investigate these differences and refine therapeutic strategies for both sexes.

## Conclusion

In conclusion, TNFSF10 orchestrates sustained central and peripheral immune responses that substantially contribute to the progression of brain pathology along with cognitive impairment in the 3xTg-AD mouse model. Thus, targeting the TNFSF10 system appears to be a powerful tool to mitigate prominent immune impairment and neuroinflammation, fostering progression of neurodegeneration. Finally, our data support the hypothesis that AD could represent a central manifestation of a disablement of the overall immune response.

## Supplementary Information

Below is the link to the electronic supplementary material.

Supplementary file1 (PDF 414 KB)

Supplementary file2 (PDF 792 KB)

Supplementary file3 (PDF 838 KB)

Supplementary file4 (PDF 1565 KB)

## Data Availability

No datasets were generated or analysed during the current study.

## Abbreviations

*3xTg-AD*: Triple-transgenic model of Alzheimer's disease
*AD*: Alzheimer’s disease
*APPswe*: Swedish mutation of the amyloid precursor protein
*Aβ*: Amyloid beta
*BBB*: Blood-brain barrier
*BCSFB*: Blood cerebrospinal fluid barrier
*C11b*: Cluster of differentiation 11b
*CD206*: Cluster of differentiation 206
*CD25*: Cluster of differentiation 25
*CD253*: Cluster of differentiation 253
*CD4*: Cluster of differentiation 4
*CD45*: Cluster of differentiation 45
*CD8*: Cluster of differentiation 8
*CD86*: Cluster of differentiation 86
*CNS*: Central nervous system
*CP*: Choroid plexus
*FoxP3*: Forkhead box P3
*GITR*: Glucocorticoid-induced TNF receptor
*IFN-γ*: Interferon-gamma
*IL-12p70*: Interleukin-12p70
*IL-17A*: Interleukin-17A
*IL-1β*: Interleukin-1β
*IL-2*: Interleukin-2
*IL-22*: Interleukin-22
*IL-23*: Interleukin-23
*IL-27*: Interleukin-27
*IL-6*: Interleukin-6
*Ly6C*: Lymphocyte antigen 6 family member C
*mAb*: Monoclonal antibody
*NK*: Natural killer
*P2RY12*: Purinergic receptor P2Y12
*PD-1*: Programmed cell death protein 1
*Psen1*: Presenilin-1
*p-Tau*: Phosphorylated-tau protein
*TNFSF10*: Tumor necrosis factor (ligand) superfamily, member 10
*TNF-α*: Tumor necrosis factor alpha
*Treg*: Regulatory T cells
*WT*: Wild-type
