# Supplementary material for: Rebalancing Immune Interactions within the Brain-Spleen Axis Mitigates Neuroinflammation in an Aging Mouse Model of Alzheimer’s Disease
Source: J Neuroimmune Pharmacol. 2025 Feb 7;20(1):15. doi: 10.1007/s11481-025-10177-7 (PMC11805801; doi:10.1007/s11481-025-10177-7)
Supplement: Supplementary file 1 — Supplementary file1 (PDF 414 KB) [file 11481_2025_10177_MOESM1_ESM.pdf]

Supplementary Figure 1

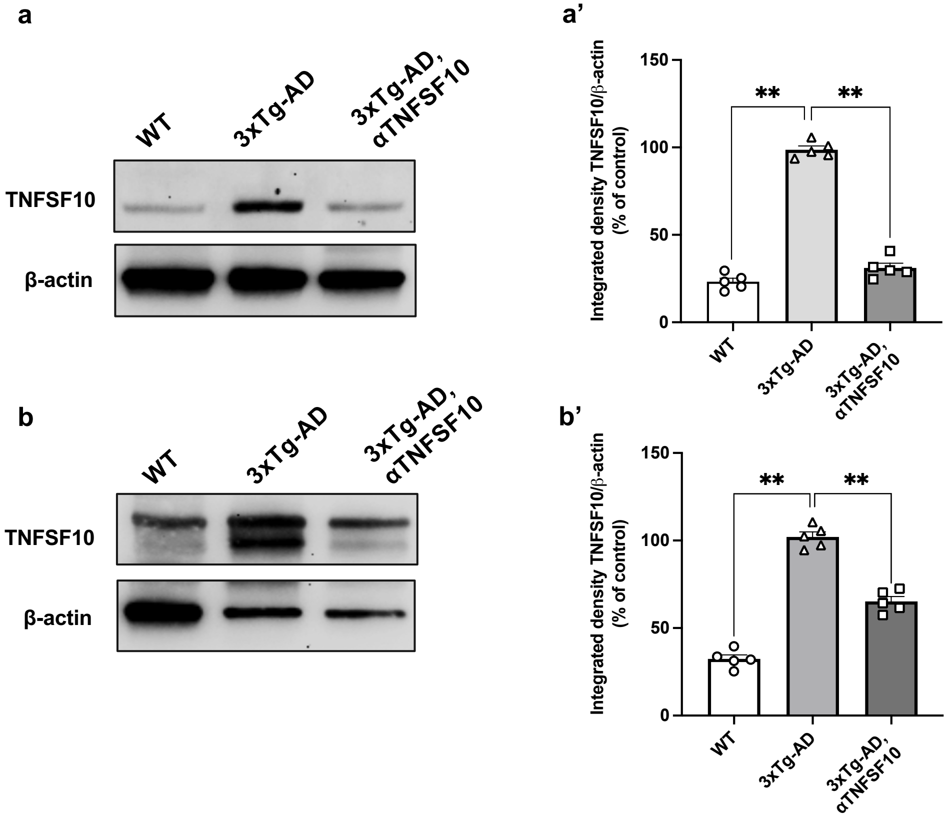

Supplementary Figure 1: (a) Western blot analysis of TNFSF10 protein expression in hippocampal lysates of wild-type and 3xTg-AD mice. (a') Respective densitometric analysis of the representative Western blot. (b) Western blot analysis of TNFSF10 protein expression in spleen lysates of wild-type and 3xTg-AD mice. (b') Respective densitometric analysis of the representative Western Blot. Data are expressed as means ± S.E.M. One-way ANOVA and the Tukey's post hoc test were used to determine statistical significance. $^{**}p < 0.01$. WT: wild-type (n = 5/group); 3xTg-AD mice (n = 5/group).
