# Supplementary material for: Rebalancing Immune Interactions within the Brain-Spleen Axis Mitigates Neuroinflammation in an Aging Mouse Model of Alzheimer’s Disease
Source: J Neuroimmune Pharmacol. 2025 Feb 7;20(1):15. doi: 10.1007/s11481-025-10177-7 (PMC11805801; doi:10.1007/s11481-025-10177-7)
Supplement: Supplementary file 2 — Supplementary file2 (PDF 792 KB) [file 11481_2025_10177_MOESM2_ESM.pdf]

Supplementary Figure 2

a

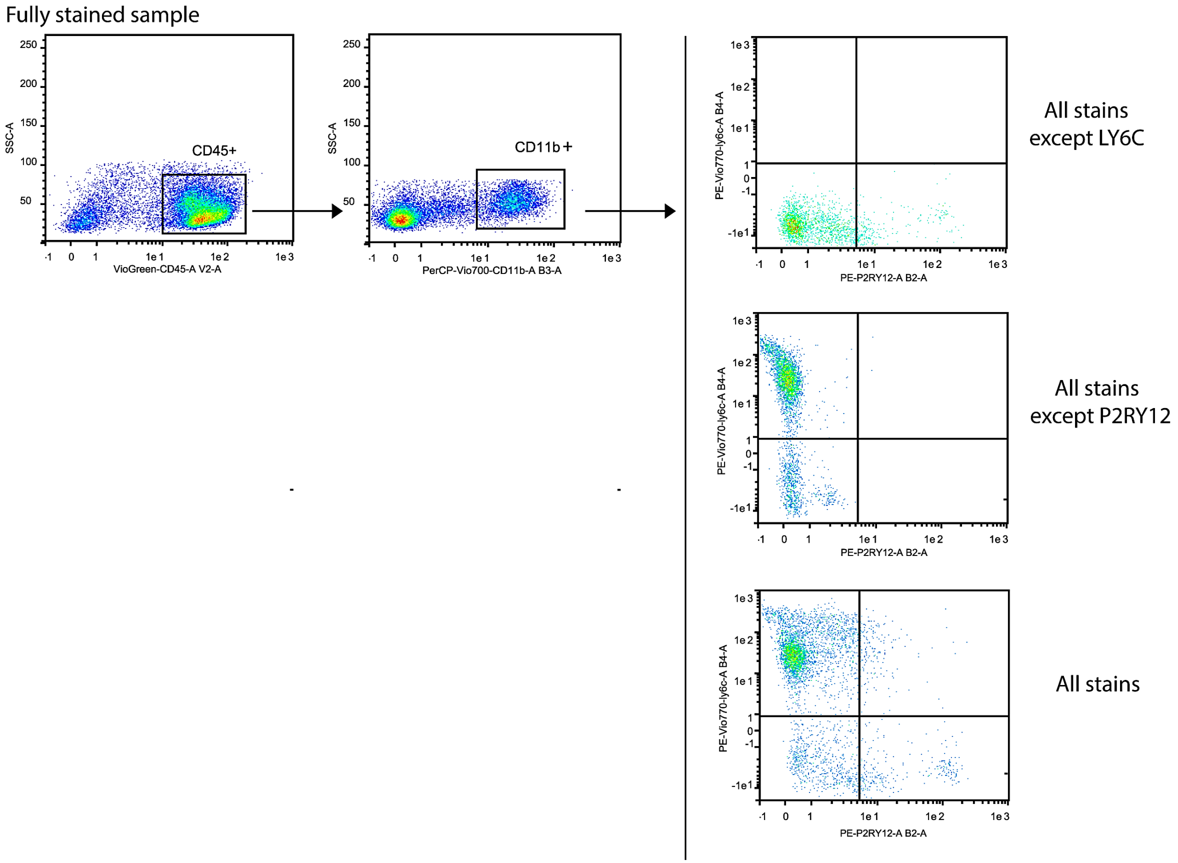

(a) Gating strategy to define proinflammatory monocytes including LY6C and P2RY12 FMO controls.

b

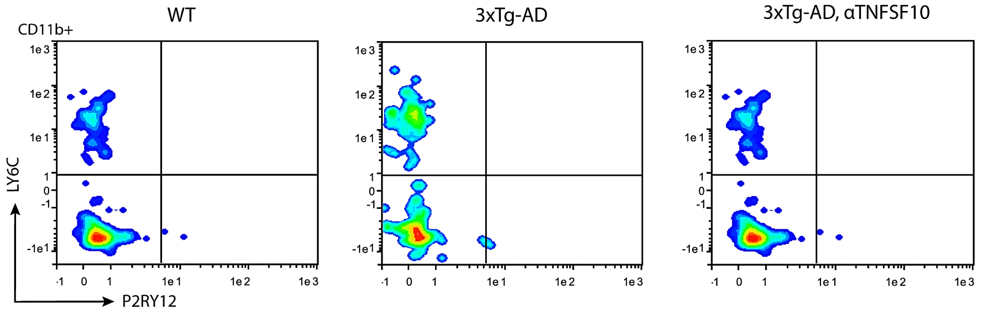

(b) Representative flow cytometry plots for each experimental group.
