# Supplementary material for: Rebalancing Immune Interactions within the Brain-Spleen Axis Mitigates Neuroinflammation in an Aging Mouse Model of Alzheimer’s Disease
Source: J Neuroimmune Pharmacol. 2025 Feb 7;20(1):15. doi: 10.1007/s11481-025-10177-7 (PMC11805801; doi:10.1007/s11481-025-10177-7)
Supplement: Supplementary file 4 — Supplementary file4 (PDF 1565 KB) [file 11481_2025_10177_MOESM4_ESM.pdf]

Supplementary Figure 4

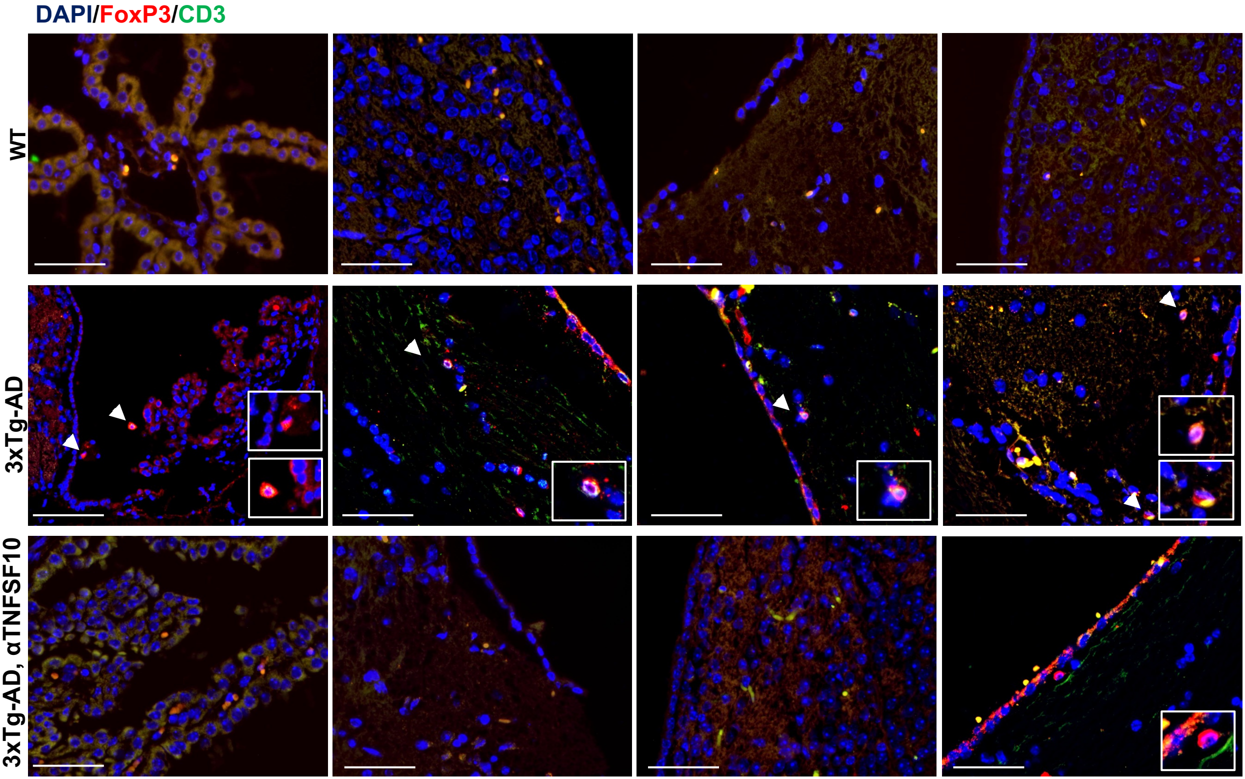

Immunohistochemical detection of CD3 and FoxP3 in the brain of wild-type and 3xTg-AD mice, treated with either vehicle or anti-TNFSF10 mAb. Original magnification 40x. Scale bars = 50 µm. The inserts in photos represent the respective areas magnified.
